# Polymer Screening for Proper Selection of Membrane Manufacturing Material with Decreased Biofouling Capacity

**DOI:** 10.3390/membranes16060188

**Published:** 2026-05-31

**Authors:** Costas Tsioptsias, Christos Manolis, Evgenios Kokkinos, Petros Samaras, Anastasios I. Zouboulis

**Affiliations:** 1 Department of Food Science and Technology, Alexandrian University Campus at Sindos, International Hellenic University, 57400 Thessaloniki, Greece; ktsiopts@ihu.gr (C.T.); dfood2008@ihu.gr (C.M.); 2 Department of Chemistry, Aristotle University of Thessaloniki, 54124 Thessaloniki, Greece; evgenios@chem.auth.gr

**Keywords:** membrane, biofouling mitigation, polymer screening, wastewater treatment, Hansen Solubility Parameters (HSPs)

## Abstract

A major limitation for the wider use of membrane-based technologies is the presence of biofouling, which is related to the decline of permeate flux, as well as the associated energy and economic costs for the necessary cleaning. In this work, the interactions and compatibility of 28 common polymeric materials with 36 potential biofoulants (categorized in six groups) is examined, based on Hansen Solubility Parameters (HSPs). Also, a simple methodology is proposed for polymer screening and comparing the suitability of 28 polymers to be used as fabrication materials or coatings, aiming to produce membranes with lower biofouling potential. The methodology gives a score to each polymer based on its interaction with water and various foulants. The screening among the commonly used polymers showed that poly (vinyl alcohol) (PVOH) is a good selection for the manufacturing of membranes, or for effective surface coating to limit biofouling, when compared to the other candidate polymers. The case of PVOH material received the highest score (11.6), while other polymers ranked with lower scores (less than 10). Its physically cross-linked nature that arises from a strong self-association pattern may also be beneficial for biofouling mitigation, since it limits the available sites for interactions (e.g., through hydrogen bonds) with the potential foulant agents. Swelling experiments on the PVOH gels with real wastewater (produced after anaerobic digestion) support the predictions for lowering the biofouling potential.

## 1. Introduction

Membrane-based technologies are used in a wide range of applications, including (among others) wastewater treatment. Specifically, a Membrane Biological Reactor (MBR) can replace the settlement tank in the commonly applied biological treatment of wastewaters, resulting simultaneously in decreasing the necessary processing time and improving the quality of treated effluent, often rendering unnecessary the common application of a tertiary treatment stage, such as disinfection/chlorination, etc. Nevertheless, the major drawback and limitation for the wider use of membrane technology are the problems related to membrane fouling, which can be summarized as follows [1,2]: (a) reduction in permeate flux over time, and (b) energy consumption and economic cost for the necessary cleaning/regenerating/reuse of membrane. Thus, the mitigation of membrane fouling has gained great research interest and various relevant approaches have been explored and proposed.

These approaches could be categorized in two broader groups: (a) those based on Fluid Mechanics options, and (b) approaches based on Chemistry aspects. Regarding the former group, lithography (including nano-imprint lithography) [1] and 3D printing [3,4,5,6] have recently attracted particular attention. Specifically, in such approaches, the main goal is to develop appropriate surface roughness and patterns in order to promote micro-turbulence around the surface elements of the membrane [2]. This turbulence can cause effective mixing and movement of suspended materials and renders more difficult their interactions and deposition onto the surface of the membrane, thus resulting in reduced fouling. Regarding the latter group of applicable technologies, the main goal is to limit the interactions between foulants and the membrane surface mainly by changing its wetting behavior [3]. This can be accomplished by modifying the surface of the membrane [7,8] via various approaches, such as formation of coatings, e.g., by addition of specific nanoparticles [9], grafting of appropriate groups [7,10,11], etc. However, several of these approaches have been questioned, regarding their cost and potential scalability [2].

Since there is a great variety of potential foulant agents in the several aqueous streams to be treated by membranes (e.g., organic, inorganic, dissolved, colloidal, etc.), as well as different mechanisms of fouling [8], the combined examination of these two broad groups is expected to increase the effectiveness of fouling mitigation. Among the major forms of fouling belongs so-called biofouling, which is believed to arise from the adsorption of Extracellular Polymeric Substances (EPSs), such as proteins and polysaccharides, onto the membrane surface, assisting the formation of biofilms, due to the adhesion and growth of microbial cells directly onto the surface of the membrane [8,12]. Therefore, when the adsorption/deposition of such substances can be limited, then biofouling will also be limited, and this is the concept behind the aforementioned surface modification approaches. However, to the best of the authors’ knowledge, the degradation products of dead cells, EPSs, etc., and their potential impact on biofouling is rarely taken into account.

Wastewaters can contain various organic substances, which have to be removed. Although the primary foulant agents are considered to be EPSs, being mainly of a protein and/or polysaccharide nature, several other organic substances, such as fats and lipids as well as the degradation products of EPSs (e.g., amino acids, peptides and sugars), can also be considered as eventual foulants. The large variety of potential foulants renders particularly complex the study of their possible interactions with the membrane’s fabrication material. The Hansen Solubility Parameters (HSPs) are regarded as a simple but powerful tool in order to study and predict the interactions between materials and their compatibility aspects, and has been used in several/different applications [13,14], including solvent screening for selective extraction [15], solvent screening for identification of low-toxicity solvents [16], solvent screening for finding green solvents for extraction purposes [17] as well as in various other applications, such as the prediction of flavor scalping from food-packaging materials [14] and migration [18], or even in glove safety issues [14]. The total solubility parameter (δ_total_) is defined as the square root of the cohesive energy density of a substance and is composed of three partial contributions: one accounting for the dispersion forces (δ_d_), the second one for the polar interactions (δ_p_) and the third one for hydrogen bonding (δ_hb_). By comparing simply two substances through their respective three partial HSPs, it can be estimated whether these substances are likely to be compatible or miscible. This concept is similar to the old rule of Chemistry that “like dissolves like” (*Similia similibus solvuntur*). Of course, the HSP approach has not had the quantitative prediction accuracy of other more precise thermodynamic models, such as COSMO-RS [19,20]. Although the HSP theory does not have the prediction power of other more complex thermodynamic models, however, it is quite simple to apply, and in contrast to other models does not require a strong background in Quantum Chemistry and Thermodynamics. In addition, it does not require the use of any special software and the HSP values of various substances can be rather easily found in the open literature. On the contrary, models such as COSMO-RS require the use of rather expensive specialized software. Moreover, the HSP calculations can be performed in a short time, while more accurate predictive models are time-consuming, requiring expensive hardware.

The use of the HSP approach as applied to polymer screening, which can be used for the construction of membranes, is very limited in the respective literature. The HSPs have been used to find the most suitable polymers to be applied as membrane materials for the recovery of Deep Eutectic Solvents (DESs), and it was reported that by the HSP approach it could be correctly predicted which polymers would be dissolved by the DESs and which solvents would exhibit poor interactions with the DESs and thus maintain their integrity [21]. To the best of the authors’ knowledge, there is only one study that has used the HSP approach to study membrane biofouling (in the case of reverse osmosis membranes) [22]. The separation of albumin protein was examined and its HSPs compared with the HSPs of eight commercial membranes by calculating the Hansen space distance R_a_. It was found that the R_a_ distance correlated with various fouling factors, such as the % irreversible fouling, and it was recommended to consider the HSP distance of the membrane and of the foulant [22]. Although these are very useful insights, however, as aforementioned only one foulant agent was examined in this case. The proper selection of membrane material should be based on the examination of interactions with more foulants. Also, the hydrophilicity of this material should also be taken into account, since it affects both permeate flux and trans-membrane pressure, independently of fouling. In summary, in the relevant literature there is a limited number of HSP studies regarding the prediction of polymer–foulant interactions and considering also the prediction of membranes’ biofouling. The existing study focuses mainly on a single typical model foulant (i.e., albumin) and ignores the membrane–water interactions.

The scope of this research is to use the HSP approach in order to study the interactions between several potential biofoulant agents with common polymeric materials that could be used for membrane fabrication and to develop a simple methodology/algorithm for polymer screening, aiming to reduce the expected biofouling issues. To the best of the authors’ knowledge there is no relevant study previously performed using this approach. Specifically, the main novelty aspects of this research are the following: (a) the predictions of polymer–foulant and polymer–water interactions based on the HSP theory, and (b) the acquired information subsequently utilized through a simple proposed algorithm for ranking various polymeric materials with respect to their suitability for using them as membranes’ construction/fabrication materials, aiming to reduce biofouling potential.

## 2. Theoretical Methodology

Several potential biofoulants were examined; these were divided into six categories, namely, carbohydrates, glycerides, amino acids, peptides and proteins, polysaccharides, and phospholipids. In addition, the affinity of examined polymeric materials with water was also examined. The concept behind the proposed methodology/“algorithm” is rather simple and similar to the previous one used for solvents’ screening, regarding the selective extraction of glycerides from microalgae [15]. The polymeric material must present high affinity for water and minimum affinity with the foulants. The high affinity with water is desired, since in the case that water is forced to pass through a hydrophobic membrane, then the trans-membrane pressure is expected to increase and the permeate flux would be decreased. On the contrary, when using a hydrophilic membrane material, higher fluxes can be achieved, as compared to the hydrophobic one, under similarly applied trans-membrane pressure. The presence of irreversible fouling suggests strong adsorption of foulants onto the membrane surface, which in turn indicates the existence of robust interactions between the foulant and the membrane material. For this reason, the membrane material must exhibit low affinity (and poor interactions) with the potential foulants.

Thus, the compatibility of 28 common polymers (potentially used as membrane construction materials) with water and with the 6 categories of eventual foulants was examined (36 substances totally considered), based on the HSP concept. The specific values of the HSPs of the polymers and foulants were retrieved from the relevant literature [13,14,23]. The HSP values are typically reported for room temperature and dry (i.e., non-hydrated) samples. For each foulant group, the average values of the HSPs were estimated from the HSP values of some representative substances. The 36 potential foulants are presented in Table S1 of Supplementary Materials. The average values and the standard deviations of the HSPs for each category are presented in Table 1.

The methodology was based on the most common approach to examine compatibility between different materials and substances, specifically, using the Hansen space distance (R_a_), which is defined as follows [13]:

(1) $$R_{a}=\sqrt{4{\left(\delta_{d1}-\delta_{d2}\right)}^{2}+{\left(\delta_{p1}-\delta_{p2}\right)}^{2}+{\left(\delta_{hb1}-\delta_{hb2}\right)}^{2}}$$

where:

δ_d_ is the dispersion HSP of substance 1 and 2 respectively, according to the subscript (in MPa^1/2^ units);

δ_p_ is the polar HSP (in MPa^1/2^);

δ_hb_ is the hydrogen bonding HSP (in MPa^1/2^).

Lower values of Ra suggest higher compatibility (thus higher probability for the dissolution, adsorption, etc., processes), whereas higher values of R_a_ suggest poor interactions and lower compatibility. The specific values of R_a_ (i.e., the meaning of lower or higher data) do not have a definite answer; however, values of R_a_ lower than 8 are generally accepted, indicating compatible substances [14]. These criteria used for the selection of appropriate polymers, i.e., values of R_a_ between a foulant category and a polymer much higher than 8 are considered as indicators of poor compatibility between the specific polymer and the respective foulant category. Therefore, the biofouling potential of this polymer for this interacting foulant category is expected to be low.

For each polymer (substance 1) and for each foulant category (substance 2) the R_a_ can be calculated from Equation (1). Also, the R_a_ between each polymer and water can be calculated with the same equation. Each polymer was ranked for each fouling potential and its suitability to be used for membrane construction based on a score, calculated by taking into account the two aforementioned aspects (i.e., the high affinity with water and the low affinity with the foulant categories).

Initially, the distance $R_{a,\;polymer/water}$ between each polymer and water and the distance $R_{a,\;polymer/foulantcategory}$ between each polymer and each foulant category was calculated from Equation 1. For each foulant category (f_c_) and polymer, the following relative distance (${RelDis}_{fc}$) can also be calculated:

(2) $${RelDis}_{fc}=\frac{R_{a,polymer/foulant\;category}}{R_{a,polymer/water}}$$

As aforementioned, the most suitable material for membrane fabrication corresponds to a polymer with minimum interactions/compatibility with all foulants and maximum compatibility with water. The main concept of the relative distance in Equation (2) is to simultaneously account for these two aspects. Thus, allowing the denominator R_a,polymer/water_ (denoting high compatibility between water and polymer) and the high numerator R_a,polymer/foulantcategory_ that will increase the value of RelDis_fc_, conforming to the previously mentioned prerequisites. Thus, when the RelDis_fc_ is high, then the satisfaction between the two sub-criteria is better (i.e., presenting higher compatibility with water and lower compatibility with foulants). The specific score of each polymer can be calculated as the sum of RelDisfc for the six foulant categories considered in this study, by also taking into account a weighting factor (w_fc_), according to the following equation:

(3) $$SCORE=\sum_{fc=1}^{6}{w_{fc}\times RelDis}_{fc}$$

The calculations using Equation (3) were performed by using equal weight, i.e., weighting factors w_fc_ = 1, for all the examined foulants. This approach was selected as the most objective for the general-purpose screening of polymers. The proposed methodology could be customized for a specific wastewater, by the identification of the most suitable material for each wastewater, based on its composition. For example, a higher w_fc_ value could be used for the foulant category in case of protein-rich wastewaters. However, for a general-purpose initial screening it seems reasonable to consider all foulant categories with equal weights. Nevertheless, a large number of potential foulants can be examined, provided that the respective HSP values are known. The specific six foulant categories selected in this work are considered representative foulants that can be typically found in many types of wastewaters, such as domestic, agricultural, food industry, algal, etc.

It should also be stressed, as mentioned in the Introduction, that the application of HSP theory is a simple, easily and rapidly applicable approach for general-purpose screening cases between different polymeric materials, e.g., helping to exclude some materials that are not suitable and focusing to the most promising ones, which should be further examined/refined. However, it does not exhibit high accuracy and thus, the ranking score derived from the HSP approach cannot be taken for granted. An uncertainty of the produced score could be derived, e.g., based on the uncertainty of HSP values of examined fouling categories; however, this analysis could be partially misleading, due to the lack of high accuracy raw values. Thus, an uncertainty analysis of the calculated scores was not performed and only the scores derived from the average values of HSP theory for the examined foulants’ categories are presented. Nevertheless, the proposed HSP methodology includes the foulant–polymer interactions, while other mechanisms possibly taking place in the overall fouling process are not accounted for, such as surface charge, surface roughness, biofilm adhesion, etc.

## 3. Materials and Methods

Poly (vinyl alcohol) (PVOH) with MW = 89,000–98,000 g/mol and >99% hydrolyzed was purchased from Sigma Aldrich, while edible (pork) gelatin films were purchased from the local market. Digestate wastewater was provided by a local biogas plant and filtered (after sedimentation) subsequently through 100, 50, 20, 10 and 5 μm filter. The Chemical Oxygen Demand (COD), total phosphorus (TP) and total nitrogen (TN) of the digestate wastewater measured photometrically with LCK kits of Hach-Lange, using the DR 2800 photometer. The Total Suspended Solids (TSS) of the digestate wastewater was measured by weighting the 1.2 μm glass fiber filter before and after the filtration of the known amount of each digestate (the drying of the filter was carried out at 105 °C).

One g of PVOH was dissolved in 10 mL of water by heating the mixture at 80 °C for 20 min. The solution was cast in a Petri dish and dried at room temperature (~12 °C) for 3 days in order to obtain the PVOH film.

The dry PVOH and gelatin films were immersed and swelled for 24 h in the digestate wastewater at room temperature (around 12 °C). Then the films were removed from the digestate and immersed in fresh distilled water for 30 min. After that, the water was removed and fresh water added. This procedure was repeated 4 times.

## 4. Results and Discussion

In Table 2, the R_a_ values of 28 potential polymers for membrane manufacturing with water, as well as with the examined six potential foulants’ categories, are presented. The appropriate evaluation of all these data is rather difficult; however there are certain cases where the physical meaning of R_a_ can be readily estimated and understood. For example, polyethylene and polypropylene (the first two polymers in Table 2) are non-polar polymers, containing only C-H bonds; thus, it is rather expected to have good compatibility with the low polarity substances, such as glycerides, and poor compatibility with water. Both of these properties are clearly observable in the respective R_a_ values, e.g., the R_a_ for the combination polyethylene-glycerides is only 6.0, whereas it is 21.7 for the polyethylene–water system. Similarly, PVOH is a highly polar polymer (the third from the end in Table 2), which is expected to have low R_a_ with water (indeed it is 6.3) and low affinity for glycerides (16.7).

**Figure 1:**
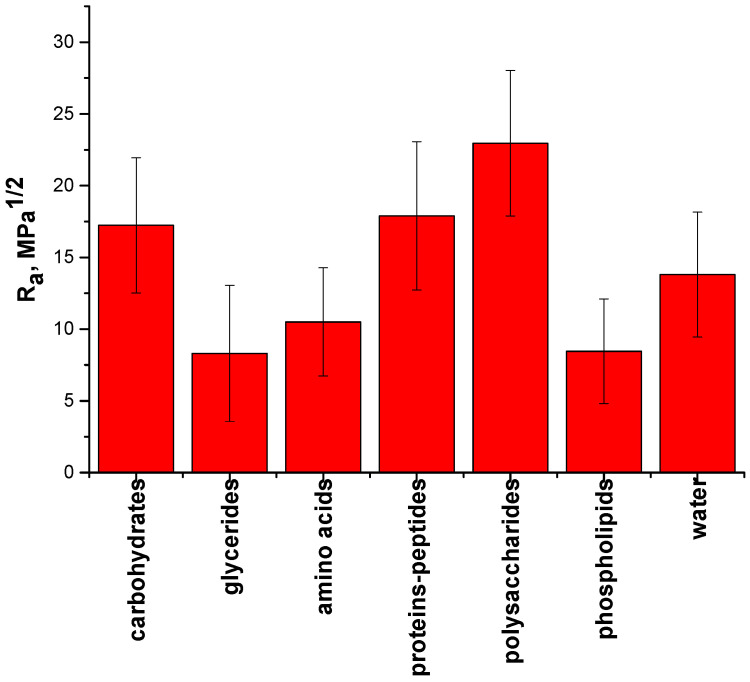
Average foulant–polymer R_a_ values for each of the six potential foulant categories.

The average values and the standard deviation for all the examined polymers and for each foulant category was calculated and presented in Figure 1.

As can be seen in Figure 1, for the vast majority of polymers the R_a_ value is rather small, for the case of lipids (glycerides and phospholipids) and for the amino acids (examined as foulants). Perhaps these substances are liberated during the lysis of cells and can play an important role in the mechanism of irreversible fouling. The previously presented data can be evaluated also in combination, through the proposed methodology/algorithm (Equations (2) and (3)) presented in Section 2. The total score and the ranking of the 28 examined polymers, as derived by Equations (2) and (3), are presented in Table 3. Based on the considered criteria (i.e., maximum affinity with water and minimum affinity for all potential foulants), it seems that PVOH is a promising candidate for membrane fabrication or coating and is worthy of further examination. Specifically, based on the R_a_ values, presented in Table 2, PVOH is expected to exhibit good compatibility with only one of the six potential foulant categories (i.e., with sugars). However, PVOH exhibits another interesting aspect, which is not taken into account in the proposed methodology, i.e., PVOH typically dissolves in water above 70 °C, while the PVOH films are physically cross-linked [24] and swell (i.e., do not dissolve) in cold water. This cross-linking arises from the strong self-association of PVOH macromolecules through hydrogen bonding (both inter- and intra-molecularly), and thus, the number of available sites for interactions (e.g., through hydrogen bonding) with the polar foulants, such as carbohydrates, should be limited. In other words, cross-linking is another interesting aspect of PVOH that could potentially contribute to reduced fouling.

The previous aspect can be supported also from the respective literature [25]; specifically, a PVOH-TiO_2_ composite was used as coating on a poly (vinylidene fluoride) (PVDF) membrane and it was found that the PVOH-coated membrane exhibited lower irreversible fouling factors, as compared to the neat PVDF membrane, when using bovine serum albumin as the foulant agent. The use of TiO_2_ was based on its photocatalytic activity, since the aim of that work was to separate and photodegrade dyes from textile industry wastewater [25]. Of course, the actual wastewaters may contain many more organic substances and potential foulants than the simple bovine serum albumin.

Among the 28 examined polymers, there were two proteins (gelatin and zein). Gelatin is more hydrophilic and soluble in water by heating (as in cold water it forms gel, similar to PVOH), while zein is insoluble in water. These two proteins exhibit quite different HSPs and for this reason their ranking is different. As can be seen, gelatin was found to be the second best choice, while the score of zein is quite lower. It should be stressed that accurate values of the HSPs regarding proteins are difficult to estimate correctly, due to their limited solubility. In addition, the heterogeneous structure of proteins also renders difficult the theoretical evaluation of HSPs, e.g., through a group contribution method.

Thus, the prediction regarding proteins should be treated only as a rough approximation. Nevertheless, gelatin and PVOH behave similarly, as they dissolve in hot water and by subsequent cooling they can form hydrogels that can be dried and turned into physically cross-linked films. These films can then be swelled in cold water. This procedure was adopted in order to provide some experimental confirmation regarding the predictions obtained for the case of PVOH. Precisely, dry PVOH and gelatin films were immersed and swollen in digestate wastewater. This procedure resulted in the production of gels with the wastewater being the gel solvent. Then, the gels were repeatedly immersed in fresh water in order to exchange the wastewater with fresh water (noting that solvent exchange is a common property of gels). It is also worth mentioning that the digestate wastewater was heavily polluted and exhibited a very high COD value (59,000 mg/L), total nitrogen content 3030 mg/L, total phosphorous content (as P-PO_4_^−3^) 284 mg/L and TSS 4.52 g/L. These parametric values showed clearly that this wastewater contained a large amount of organic substances. In addition, it was expected that the remaining anaerobic digestion (digestate) would also contain various organic substances, dead and alive bacteria, products of bacterial lysis, etc. Thus, this wastewater can be considered to be representative of a typical “strong” wastewater with increased fouling potential, when treated by membranes.

**Figure 2:**
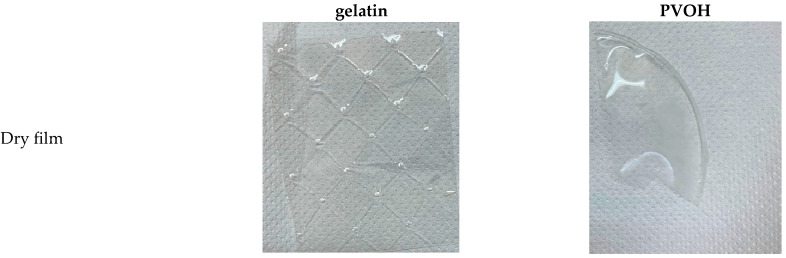

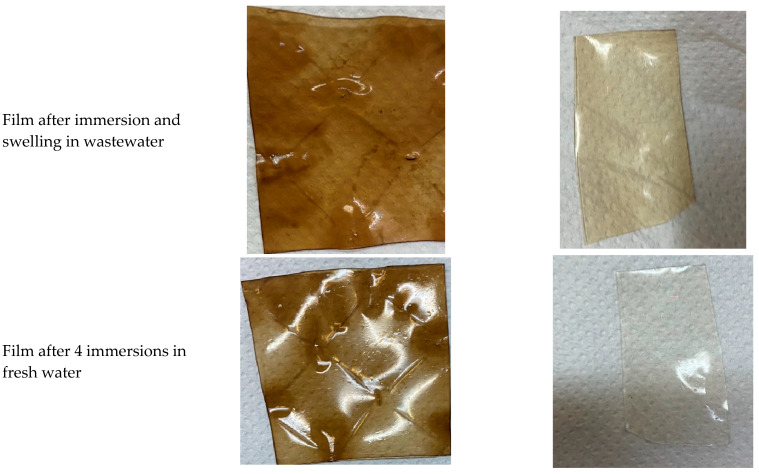
Photographs of the PVOH and gelatin films in the dry state, after swelling in wastewater and after being immersed/washed in fresh water 4 times.

In Figure 2, photographs of the PVOH and gelatin films in the dry state, after immersion and swelling in the digestate wastewater, and after washing with water (by being immersed in fresh water four times) are presented.

As can be observed in Figure 2, initially the dry films were transparent and colorless. After being immersed, the films absorbed wastewater, resulting in their swelling, due to the penetration of dissolved organic matter along with water into the films. Thus, after immersion in wastewater the samples were swollen and exhibited a dark brown color. By comparing the color of the gels, it seems that the absorption of organic matter in the case of PVOH was lower than that of gelatin, since the PVOH sample exhibited a less dark color than the gelatin sample. This suggests that poorer interaction between the organic substances of the wastewater and the PVOH took place. Most importantly, the absorption of such substances in the case of PVOH is quite reversible, since by repeatedly immersing the contaminated colored gel in fresh water, the color of the PVOH gel was gradually decreased after four immersions, as can be seen in the images of Figure 2. On the contrary, in the case of the gelatin gel no signs of reversibility could be detected and the color remained unchanged even after four immersions in fresh water. This observation suggests that in the case of gelatin the polymer–foulant interactions could be thermodynamically favored and thus are difficult to reverse. For the case of PVOH, as suggested by the proposed methodology, the interactions with various organic substances may be poorer, while the PVOH–water interactions could be stronger (i.e., thermodynamically favorable), which could provide a plausible explanation for the reversibility of the absorption of wastewater in PVOH.

**Figure 3:**
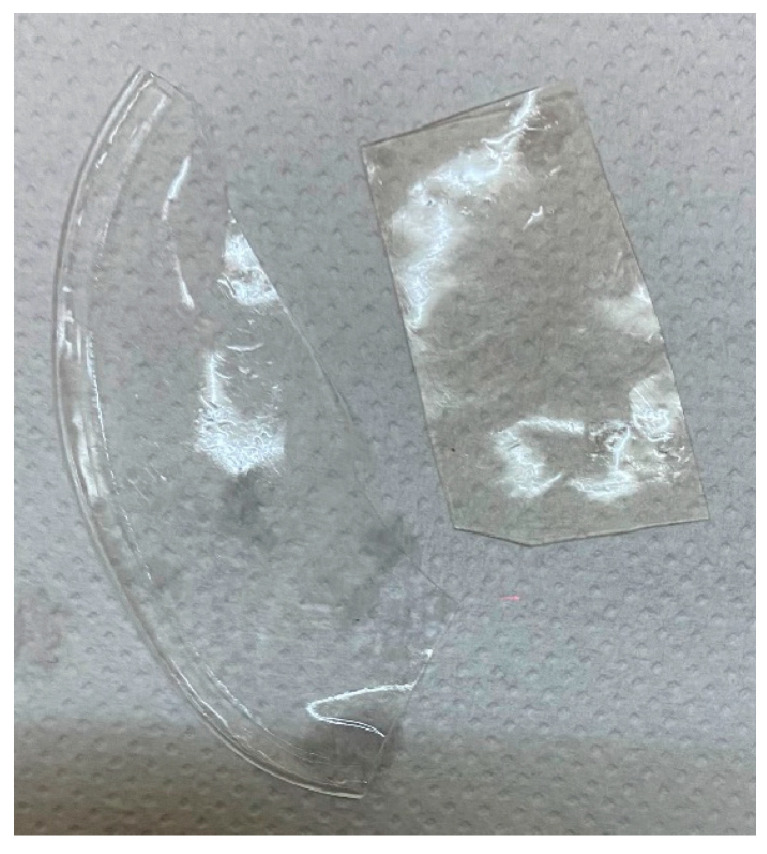
PVOH film swollen in water (left) and PVOH film swollen in wastewater and immersed in fresh water 4 times (right).

It should be stressed however, as shown in Figure 3, that the color was not fully removed by the applied four washings in the case of PVOH. In Figure 3 photographs of the washed PVOH gel along with a gel swelled in water are presented. It can be seen that in the case of the wastewater-swelled gel the color, even after four washings, has a slight brownish hue. Thus, the reversibility was not accomplished to 100%, but in any case, the absorption of the wastewater from PVOH can be reversed to quite a large extent. This suggests that an easy cleaning/regeneration procedure may be feasible.

Also, it is worth discussing the issue of PVOH biodegradation. Obviously, the biodegradation potential of PVOH could limit its wide utilization as a construction material or as a surface coating for membranes, when those would be applied in wastewater treatment. However, the application of chemical cross-linking and/or encapsulation of disinfectants could overcome (partly) this disadvantage. In addition, both of these treatments could contribute to other aspects as well. For example, the chemical cross-linking can enhance the mechanical integrity of the polymer and its chemical stability against the use of cleaning agents. As discussed above, the chemical cross-linking can also “block” some groups and render them unavailable for interacting strongly with the surrounding molecules. Also, the presence of disinfectants can protect the PVOH from biodegradation, but could also be beneficial for mitigating biofilm formation onto the surface of the membrane. PVOH is hard to extrude, but it can be processed from aqueous solutions. It can be used as 3D-printing material [26]. Also, it can very easily be applied as appropriate coating onto an existing membrane.

In addition, the placement of some other polymers in the top-ranked positions is of interest and is discussed with more details in the following. The Poly (ethylene vinyl) alcohol is structurally close to PVOH and it may exhibit high antifouling potential, similarly to PVOH, requiring further investigation, noting that it is already used for such applications [27,28]. PVDF was found to be among the highest ranking positions, noting also that several commercial membranes are based on PVDF [29], considered as a widely accepted suitable material for membrane fabrication. From the values of Ra presented in Table 2 it seems that PVDF has sufficient compatibility with water and poor interactions with various potential foulants, such as proteins and sugars. The primary drawback of PVDF is its compatibility with amino acids and lipids, especially with phospholipids; it could be stated that PVDF should not be preferred for effluents that are rich in oils and fats. Poly (acrylonitrile) (PAN) is another common material for membrane fabrication [30], which is ranked among the top positions of the relevant list, based on the proposed methodology. Considering the Ra values in Table 2 it seems that PAN exhibits poor compatibility with all foulants, but also poor compatibility with water. Polyether sulfone (PES) is also widely used for membrane fabrication [31]; from Table 2 it seems that it has similar drawbacks as PVDF, i.e., moderate compatibility with amino acids and lipids and lower compatibility with water than PVDF. Thus, it seems that the PES membranes may not be ideal for wastewaters rich in amino acid and oils. Finally, the ranking of gelatin may be misleading, since the evaluation of its HSPs is undermined by its limited solubility.

Thus, it is evident that the proposed methodology could be used not only for wastewater applications, but also for other membrane-based applications as well, such as the membranes used for the fractionation of oligomers, e.g., sugars, amino acids/peptides used in the food industry, etc. As mentioned in the Section 2, depending on the composition of the treated liquid, weight factors can also be properly applied to modify the score of each polymer. From the data presented in Table 2, useful insights can be provided as a general guide for excluding some polymeric materials and focusing on others, depending on the specific characteristics of the liquid phase. For example, a PES membrane does not seem to be the best choice for amino acid separation applications and other more suitable polymeric material should be identified.

Finally, it should be mentioned that six foulant categories and 28 polymers were examined in this study. Provided that the HSP values of the examined cases are known, the proposed approach can be directly applied for the preliminary screening of a large number of polymeric materials’ potential fabrication for membranes under different foulants’ presence.

## 5. Conclusions

The compatibility of 28 common polymers with various biofoulant categories was examined in this research by applying a simple methodology/algorithm, based on their HSP values. The proposed algorithm takes into account the occurrence of high compatibility between polymers and water (by enabling the wetting of the surface and the flow of water) and the low compatibility with various foulants (resulting in poor interactions and adsorption on the membrane’s surface). Among the 28 polymers, the hydrophilic polymer PVOH was found to be the optimal choice for membrane fabrication, or applied as coating, in terms of reduced biofouling issues. The swelling experiments of PVOH with real and heavily polluted digestate wastewater seemed to confirm these predictions. Although the PVOH was swelled by the wastewater, this procedure could be rather easily reversed. However, this was not the case for gelatin, suggesting poor interaction of PVOH with the wastewater and stronger interaction with the fresh water. The (physically) cross-linked nature of PVOH could also be beneficial for the mitigation of fouling, since the cross-linking arises from strong self-association and limits the available sites for interactions (e.g., through hydrogen bonding) with the polar foulants. Chemical cross-linking and the encapsulation of disinfectants could further assist in the mitigation of fouling along with better protection from biodegradation.

## Figures and Tables

**Table 1:** The average values and standard deviations of the HSPs for the six considered categories of potential foulants.

Foulant Category	δ_d_, MPa^1/2^	δ_p_, MPa^1/2^	δ_hb_, MPa^1/2^
Carbohydrates	17.1 ± 1.3	14.7 ± 2.8	23.0 ± 3.5
Glycerides	16.1 ± 0.1	4.3 ± 0.4	7.5 ± 3.7
Amino acids	17.9 ± 1.0	9.3 ± 3.3	16.3 ± 3.6
Proteins–Peptides	19.3 ± 0.8	21.1 ± 4.5	19.3 ± 2.3
Polysaccharides	24.3 ± 0.0	19.9 ± 0.0	22.5 ± 0.0
Phospholipids	16.6 ± 0.8	8.6 ± 3.2	12.5 ± 5.4

**Table 2:** R_a_ values for the 28 examined polymers with water and with the 6 potential foulant categories; all values are presented in MPa^1/2^ units.

Polymer	Carbohydrates	Glycerides	Amino-acids	Proteins–Peptides	Polysaccharides	Phospholipids	Water
Polyethylene	24.5	6.0	16.1	26.5	31.2	12.5	21.7
Polypropylene	26.5	8.6	17.9	28.0	31.9	14.6	23.4
Polystyrene	22.7	6.6	14.3	23.3	27.5	11.1	18.9
Polyvinylchloride	21.2	8.2	13.2	20.6	24.8	10.5	16.5
Polyacrylonitrile	17.5	16.0	12.5	13.8	15.1	13.2	12.0
Polymethylmethacrylate	18.6	8.3	11.4	17.7	22.8	8.6	13.6
Polyethylmethacrylate	19.7	7.1	12.3	19.3	25.0	8.8	14.9
Polycarbonate	18.5	4.5	10.0	19.7	24.3	7.0	15.0
Polycaprolactone	17.6	3.4	9.0	19.6	24.4	5.9	14.8
Polyvinylacetate	22.8	5.0	14.2	24.5	28.9	10.8	19.7
Polyamide	9.7	9.4	2.1	12.7	18.8	2.9	7.7
Polyethyleneterephthalate	18.5	4.8	10.1	19.5	24.2	7.1	14.9
Polyvinylbutyral	14.7	7.5	6.1	17.8	21.5	5.7	13.3
Polyvinylidenefluoride	13.0	8.5	7.0	13.5	20.6	4.3	8.6
Polyphenyleneoxide	18.6	3.9	10.0	21.1	25.3	7.3	16.3
Polyurethane	19.4	7.1	11.8	19.0	24.3	8.6	14.6
Polysulphone	18.7	1.9	10.9	20.7	26.9	6.6	15.7
Polysilicone	23.2	5.1	14.8	24.6	29.5	11.1	19.9
Polyethersulphone	15.9	8.9	8.8	15.1	20.0	7.0	10.9
Polyoxymethylene	14.3	5.9	6.7	15.7	21.8	3.0	10.8
Polyvinylpyrrolidone	10.5	8.8	2.0	14.2	19.6	3.1	9.4
Polyethylene oxide	18.6	6.5	11.5	18.6	24.8	7.7	14.0
Polypropylene oxide	17.0	4.8	9.7	18.1	24.5	5.6	13.2
Polyvinylalcohol	7.1	16.7	9.9	9.5	19.4	10.6	6.3
Ethylenevinylalcohol	10.6	10.4	6.9	12.7	21.2	5.0	7.7
Polylacticacid	17.9	7.6	10.4	17.4	22.4	7.7	13.1
Zein	12.2	18.2	9.5	12.8	11.2	13.5	11.6
Gelatin	13.0	22.7	15.1	4.6	11.0	17.1	7.9

**Table 3:** The total score and ranking of the examined 28 polymers, regarding their antifouling potential and their suitability for membrane fabrication.

Polymer	Short Name	Total Score	Ranking
Polyvinylalcohol	PVOH	11.69	1
Gelatin		10.56	2
Ethylenevinylalcohol	EVOH	8.69	3
Polyvinylidenefluoride	PVDF	7.74	4
Polyacrylonitrile	PAN	7.35	5
Polyamide	nylon 66	7.22	6
Polyethersulphone		6.92	7
Zein		6.71	8
Polymethylmethacrylate	PMMA	6.45	9
Polylacticacid	PLA	6.37	10
Polyoxymethylene	PON	6.26	11
Polyethylene oxide (poly ethylene glycol)	PEO, PEG	6.25	12
Polyvinylpyrrolidone	PVP	6.20	13
Polyethylmethacrylate	PEMA	6.19	14
Polyurethane	PU	6.15	15
Polypropylene oxide (poly propylene glycol)	PPO, PPG	6.04	16
Polyvinylchloride	PVC	5.97	17
Polyethyleneterephthalate	PET	5.66	18
Polystyrene	PS	5.60	19
Polycarbonate	PC	5.59	20
Polyvinylbutyral	PVB	5.50	21
Polypropylene	PP	5.46	22
Polysulphone		5.45	23
Polysilicone		5.45	24
Polycaprolactone	PCL	5.39	25
Polyethylene	PE	5.39	26
Polyvinylacetate	PVAc	5.38	27
Polyphenyleneoxide	PPO	5.28	28

## Data Availability

Data contained in the article and Supplementary Materials.

## Supplementary Materials

The following supporting information can be downloaded at https://www.mdpi.com/article/10.3390/membranes16060188/s1, Table S1: The six categories and 36 potential foulants that were considered.
