# Supplementary material for: Polymer Screening for Proper Selection of Membrane Manufacturing Material with Decreased Biofouling Capacity
Source: Membranes (Basel). 2026 May 31;16(6):188. doi: 10.3390/membranes16060188 (PMC13302931; doi:10.3390/membranes16060188)
Supplement: Supplementary file 1 [file membranes-16-00188-s001.zip › membranes-4232902-supplementary.pdf]

Supporting information for

# Polymer screening for proper selection of membrane's manufacturing material with decreased bio/fouling capacity

**Costas Tsioptsias [1], Christos Manolis [1], Evgenios Kokkinos [2], Petros Samaras [1,*] and Anastasios I. Zouboulis [2,**]**

[1] Department of Food Science and Technology, Alexandrian University Campus at Sindos, International Hellenic University, 57400, Thessaloniki, Greece;
[2] Department of Chemistry, Aristotle University of Thessaloniki, 54124, Thessaloniki, Greece.
* Correspondence: samaras@ihu.gr (P.S.)
** Correspondence: zoubouli@chem.auth.gr (A.Z.)

**Table S1.** The six categories and 36 potential foulants that were considered.

| |
|---|
| **carbohydrates** |
| Lactose |
| Dextrose |
| Sorbitol |
| Sucrose |
| Dextro-Xylose |
| |
| **fats and lipids** |
| Glyceryl Monostearate |
| Glyceryl Monooleate |
| Glyceryl Trioleate |
| |
| **amino acids** |
| d-p-Hydroxyphenylglycine |
| Glycine |
| Phenylglycine |
| n-Leucylglycine |
| l-Proline |
| Alanine |
| l-Cysteine |
| Laevo-Histidine |
| Laevo-Arginine |
| l-Asparagine |
| Lysine |
| Laevo-Aspartic Acid |
| Laevo-Glutamine |
| l-Phenylalanine |

| |
|---|
| Methionine |
| Serine |
| l-Tryptophan |
| l-Tyrosine |
| Threonine |
| Isoleucine |
| Leucine |
| Valine |
| |
| **peptides-proteins** |
| n-(n-Glycylglycyl)Glycine |
| BSA (Bovine serum albumin) |
| |
| **polysaccharides** |
| Dextran C (= amorphous cellulose) |
| |
| **phospholipids** |
| Phosphatidylcholine |
| Phosphatidylethanolamine |
| Phosphatidylserine |
